# Porous Surface Modified Bioactive Bone Cement for Enhanced Bone Bonding

**DOI:** 10.1371/journal.pone.0042525

**Published:** 2012-08-08

**Authors:** Qiang He, Huiling Chen, Li Huang, Jingjing Dong, Dagang Guo, Mengmeng Mao, Liang Kong, Yang Li, Zixiang Wu, Wei Lei

**Affiliations:** 1 Institute of Orthopaedics, Xijing Hospital, Fourth Military Medical University, Xi’an, Shaanxi, People’s Republic of China; 2 Department of Health Service, School of Public Health and Military Preventive, Fourth Military Medical University, Xi’an, Shaanxi, People’s Republic of China; 3 Department of General Dentistry, School of Stomatology, Fourth Military Medical University, Xi’an, Shaanxi, People’s Republic of China; 4 State Key Laboratory for Mechanical Behavior of Materials, School of Materials Science and Engineering, Xi’an Jiaotong University, Xi’an, Shaanxi, People’s Republic of China; 5 Department of Oral and Maxillofacial Surgery, School of Stomatology, Fourth Military Medical University, Xi’an, Shaanxi, People’s Republic of China; University of Minho, Portugal

## Abstract

**Background:**

Polymethylmethacrylate bone cement cannot provide an adhesive chemical bonding to form a stable cement-bone interface. Bioactive bone cements show bone bonding ability, but their clinical application is limited because bone resorption is observed after implantation. Porous polymethylmethacrylate can be achieved with the addition of carboxymethylcellulose, alginate and gelatin microparticles to promote bone ingrowth, but the mechanical properties are too low to be used in orthopedic applications. Bone ingrowth into cement could decrease the possibility of bone resorption and promote the formation of a stable interface. However, scarce literature is reported on bioactive bone cements that allow bone ingrowth. In this paper, we reported a porous surface modified bioactive bone cement with desired mechanical properties, which could allow for bone ingrowth.

**Materials and Methods:**

The porous surface modified bioactive bone cement was evaluated to determine its handling characteristics, mechanical properties and behavior in a simulated body fluid. The in vitro cellular responses of the samples were also investigated in terms of cell attachment, proliferation, and osteoblastic differentiation. Furthermore, bone ingrowth was examined in a rabbit femoral condyle defect model by using micro-CT imaging and histological analysis. The strength of the implant–bone interface was also investigated by push-out tests.

**Results:**

The modified bone cement with a low content of bioactive fillers resulted in proper handling characteristics and adequate mechanical properties, but slightly affected its bioactivity. Moreover, the degree of attachment, proliferation and osteogenic differentiation of preosteoblast cells was also increased. The results of the push-out test revealed that higher interfacial bonding strength was achieved with the modified bone cement because of the formation of the apatite layer and the osseointegration after implantation in the bony defect.

**Conclusions:**

Our findings suggested a new bioactive bone cement for prosthetic fixation in total joint replacement.

## Introduction

Tight fixation between polymethylmethacrylate (PMMA) bone cement and bone is of great importance for a successful outcome of total joint replacement. The fixation strength of PMMA cement to bone is primarily dependent on mechanical interlocking [1], [2]. To achieve interlock, the bone surface must be rough and irregular. Although a good fixation of PMMA cement can be achieved by interlocking into pores of implants and bone [3], a fibrous tissue layer always intervenes between cement and bone [4], [5]. The layer is known as the weak-link zone and can lead to loosening of the prosthesis [6].

Several strategies are employed to improve PMMA based cement-bone interactions. One of the strategies attempted is to develop bioactive bone cements by incorporation of all sorts of bioceramics into PMMA bone cement. Various bioceramics have been studied, including bone, glass, and calcium phosphate compounds, such as hydroxyapatite and tricalcium phosphates [7]–[10]. The bioactive bone cements can bond directly to the bone, but the pre-clinical results are far from satisfactory. The addition of excess amounts of ceramic power to the PMMA cement adversely affects the mechanical and handling properties [11]–[12]. Moreover, bone resorption is observed after implantation in the bioactive bone cement group, which will gradually compromise fixation. This is because weakness of the calcium phosphorous layer formed on the surface of the bioactive bone cement results in particles of wear debris and stimulates bone resorption [13]. Another strategy is to provide porosity in PMMA bone cement with the addition of carboxymethylcellulose (CMC) [14], alginate [15] and gelatin microparticles (GMPs) [16]. The porous PMMA can promote ingrowth of soft and hard tissue into the material, thereby creating more interlocking and the anchorage of the PMMA. However, the mechanical properties of the porous PMMA are too low to be used in orthopedic applications. Previous studies revealed that bone ingrowth into bone cement could decrease the possibility of bone resorption and promote the formation of a stable interface [17]. Therefore, bone ingrowth into bioactive bone cement is of importance in developing adequate initial fixation. Recently, Lye KW et al proposed a porous PMMA cement incorporated with β-TCP particles, but the addition of β-TCP did not convey any advantage in terms of increase in bone formation and ingrowth due to the way the β-TCP particles were included into the PMMA matrix [18]. Scarce literature is reported on bioactive bone cements that allow bone ingrowth.

**Table 1:** Compositions of the bone cements prepared.^a^

	PSB bone cement	PMMA bone cement
Solid component		
PMMA	48.5	98.5
Glass	40.0	0
CS	10.0	0
BPO	1.5	1.5
Liquid component		
MMA	99.0	99.0
DMPT	1.0	1.0

a by weight ratio (wt%) of solid component and liquid component, respectively.

**Table 2:** Values of curing parameters of different cements (n = 6).

Cement	Dough time(min)	Setting time(min)
PSB bone cement	3.8±0.4	12.7±0.3*
PMMA bone cement	3.0±0.2	8.6±0.3

* , *P*<0.05 compared to PMMA bone cement.

The objective of this study was to prepare a bioactive bone cement with desired mechanical properties, which could allow for bone ingrowth. The bioactive bone cement consisted of low content of bioactive glass as bioactive fillers, chitosan particles as porogen, and polymethylmethacrylate as the matrix. Chitosan is a safe ingredient that is biodegradable and environmentally biocompatible. The porous surface structure obtained by the degradation of the chitosan particles will promote bone ingrowth and improve the interfacial bonding strength. In addition, with the low content of bioactive fillers, proper handling characteristics, adequate mechanical properties and direct bone contact can be achieved. In the present study, the porous surface modified bioactive bone cement was evaluated to determine its handling characteristics, mechanical properties and behavior in a simulated body fluid (SBF). The in vitro cellular responses of the samples were also investigated in terms of cell attachment, proliferation, and osteoblastic differentiation. Furthermore, bone ingrowth was examined in a rabbit femoral condyle defect model by using micro-CT imaging and histological analysis. The strength of the implant-bone interface was also investigated by push-out tests.

## Materials and Methods

### 1. Preparation of the Porous Surface Modified Bioactive Bone Cement

The raw materials used for the preparation and composition of the bone cement are listed in Table 1. Two types of cements, the porous surface modified bioactive bone cement (designated as PSB bone cement) and PMMA bone cement, were prepared. The PMMA bone cement was used as a control material. The bioactive glass (glass) was glass 45S5 particles, which were pulverized from NovaBone® product (LLC, Alachua, USA). This glass has a composition of 45 wt% SiO_2_, 24.5 wt% CaO, 24.5 wt% Na_2_O and 6 wt% P_2_O_5_. Chitosan (CS) was purchased from Biochemical Medicine Plant of Qingdao (Qingdao, China) and obtained by the method described in a previous study [19]. The average molecular weight of CS particles was 2000–3000 g/mol, and the extent of deacetylation was approximately 85%. The microstructures of the CS and glass particle were examined by scanning electron microscopy (SEM, Hitachi S-2400, Japan). Pure PMMA powder was obtained from Industrias Quirúrgicas de Levante (Asturias, Spain). Methyl methacrylate (MMA, Acros Organics, Fisher Scientific, UK) was used as received. Benzoyl peroxide (BPO, Fluka, Sigma–Aldrich, UK) initiator and N,N-dimethyl-p-toluidine (DMPT, Fluka) activator were used as received for the polymerization reaction. The solid component consisted of PMMA beads, glass particles, CS particles, and BPO as the initiator. The liquid component consisted of MMA monomer and DMOH as activator of reduced toxicity. The paste was prepared by mixing the powder with the liquid using a solid: liquid mass ratio of 2∶1 under ambient conditions at room temperature.

**Figure 1:**
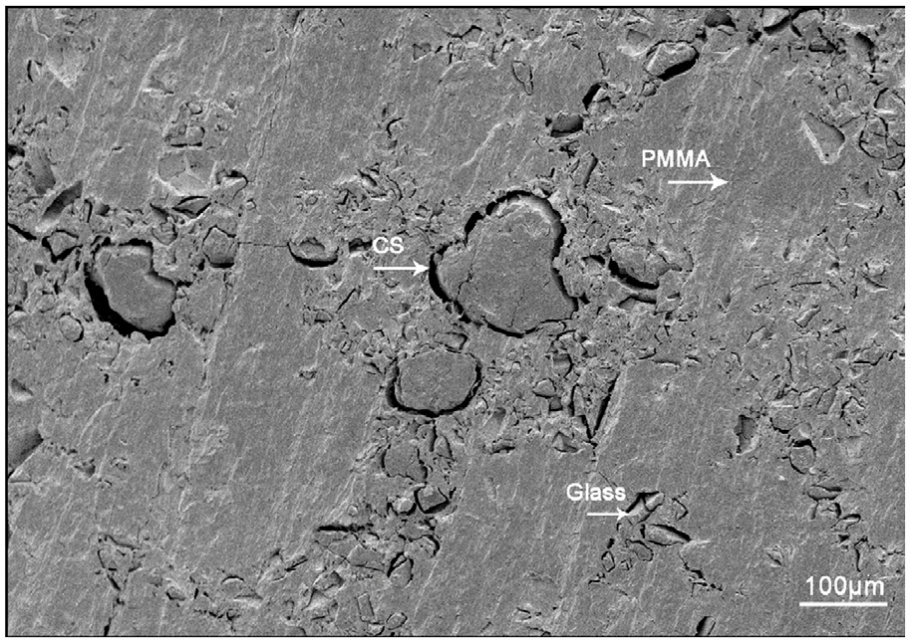
SEM images of the PSB bone cement fresh prepared. The glass and CS particles were uniformly distributed in the polymeric matrix for dry cement samples.

### 2. Characterisation of the PSB Bone Cement

Sample of the PSB bone cement was gently washed with distilled water three times to remove the polymer on the surface and dried in a fume hood overnight. Then the sample surface was examined with scanning electron microscopy (SEM, Hitachi S-2400, Japan).

### 3. Curing Parameters

In order to compare curing parameters of the PSB bone cement with those of the PMMA bone cement, dough time and setting time were measured. Dough time and setting time were determined according to International Standard Specification (ISO 5833) [20]. Dough time is defined as the time at which the cement mass no longer adheres to a surgically gloved finger. The setting time of cement sample was measured using a vicat needle (SS-S-403, Shinohara Manufacturing Co., Ltd., Tochigi, Japan). The cement paste was mixed for 3 min and cast into a cylindrical mold made of polytetrafluoroethylene (inner diameter = 6 mm, inner depth = 6 mm). The vicat needle with cross-sectional area of 1 mm^2^ was gently placed on the surface of the molded cement for time intervals of 30 s. The time required for the needle trace to disappear after placing the vicat needle on the surface was measured under ambient conditions of temperature = 21–22°C and humidity = 26–28%. The setting time was defined as the point when the vicat needle no longer gave a trace in the cement surface [21].

**Figure 2:**
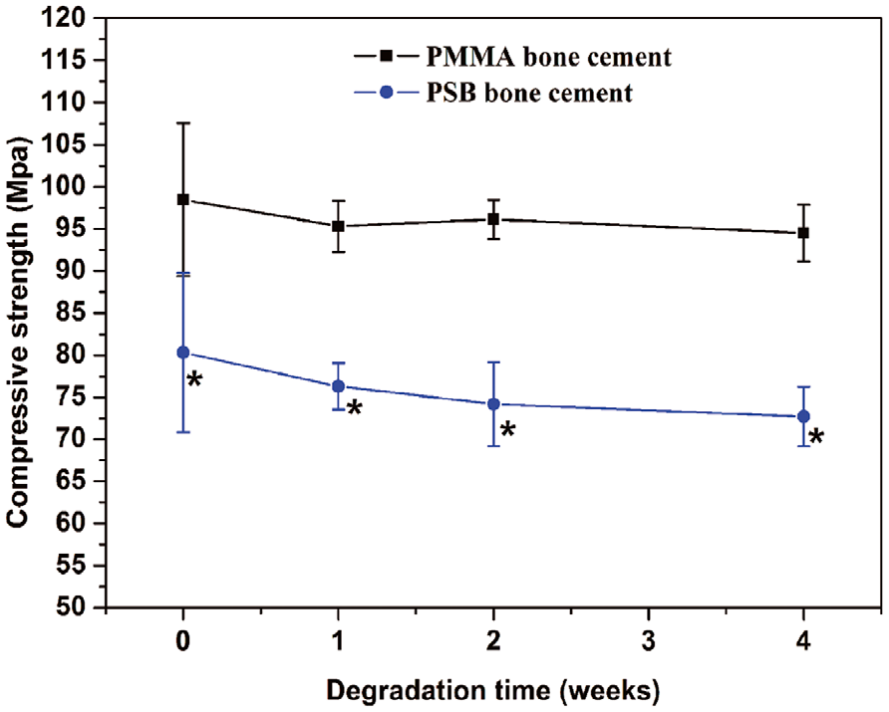
The degradation of mechanical properties of the PMMA and PSB bone cement. A significantly lower compressive strength (*P<*0.05) was observed for the PSB bone cement compared to the PMMA bone cement at each degradation time.

### 4. Compressive Strength and Degradation Testing

For the compressive strength test, cylindrical samples of cured cement were prepared with diameters of 6 mm and lengths of 12 mm [22]. Then samples were immersed in 15 ml phosphate buffered saline (PBS) at 37°C and kept for 4 weeks. Every 2 days, the immersion solution was renewed. Six samples were taken out and dried for measurements at 1, 2, and 4 weeks, respectively. The compressive load was applied at a loading rate of 0.01 mm/s using an MTS materials testing system (MTS 858 Bionix machine, MTS System Inc., Minneapolis, MN). The compressive strength was calculated from the compressive load and geometric area of the samples.

### 5. Behavior of the Sample in SBF

The PSB bone cement was molded into rectangular shapes of 5 mm×5 mm×5 mm. Then the prepared specimens were soaked in 10 ml of SBF at 37°C. The SBF solution was prepared by dissolving NaCl, NaHCO_3_, KCl, K_2_PO_4_•3H_2_O, MgCl_2_•6H_2_O, 1.0 M HCl, CaCl_2_, Na_2_SO_4_ and (HOCH_2_)_3_CNH_2_ in ultrapure water [23]. After 7 days, the specimens were removed from the solution, and gently washed with distilled water. Surface changes on the specimens were characterized by scanning electron microscopy (SEM, Hitachi S-2400, Japan) and an energy dispersive spectrometer (EDS, Falcon, USA).

**Figure 3:**
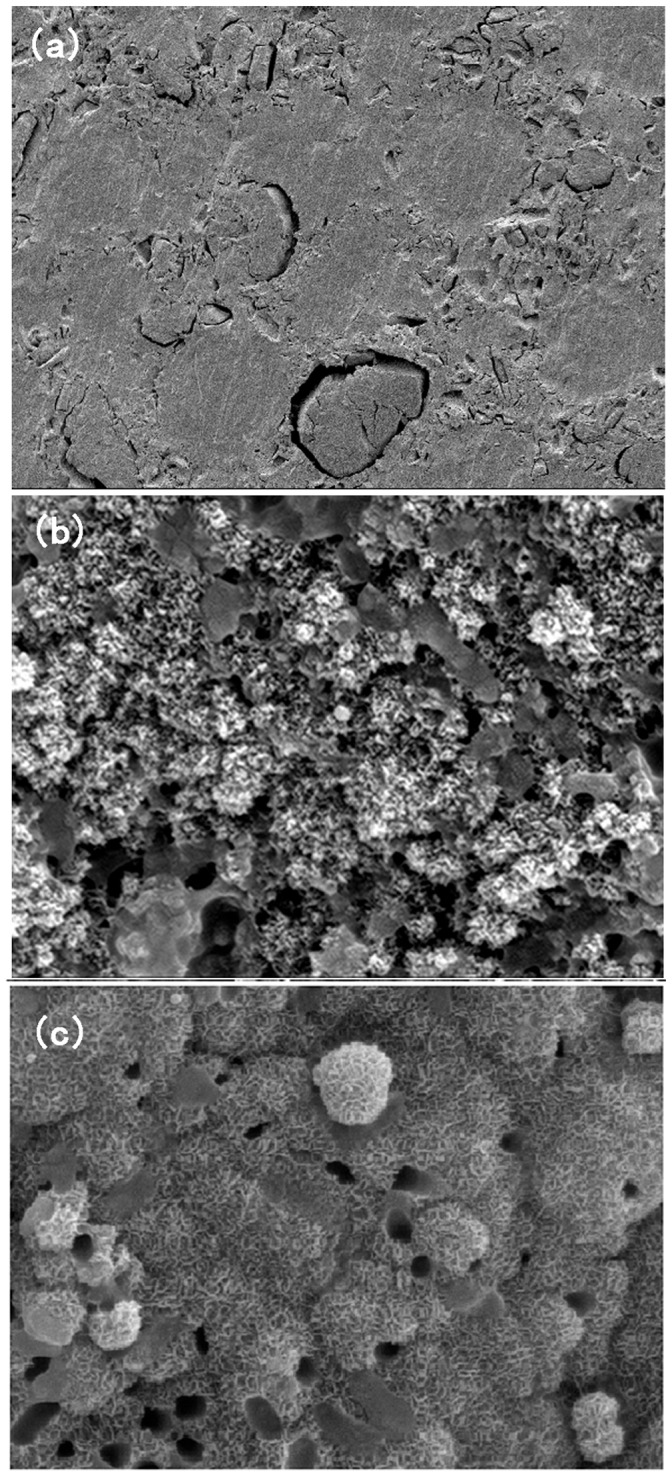
SEM photographs of the surface of PSB bone cement before and after soaking in SBF for different periods. (a) Samples before soaked in SBF. (b) Samples after soaking in SBF for 3 days. (c) Samples after soaking in SBF for 7 days. After 3 days, the surface changed considerably (as compared to the non-immersed specimens), but no deposits were visible even at high magnifications. Apatite crystals of needle-like morphology were observed on the surface of the cement sample soaked in SBF for 7 days.

### 6. *In vitro* Cellular Response

#### 6.1 Cell culture

MC3T3-E1 subclone 4 mouse preosteoblast cells (American Type Culture Collection) were grown in culturing medium consisting of a-MEM (Hyclone, USA) containing 100 units per ml penicillin and 100 µg per ml streptomycin and supplemented with 10 vol. % fetal bovine serum (Hyclone, USA) at 37°C with 5% CO_2_. Osteogenic medium was prepared by adding 10 mM b-glycerophosphate (Sigma–Aldrich, UK) and 50 mg/ml ascorbic acid (Sigma–Aldrich, UK) into culturing medium for the alkaline phosphatase (ALP) test.

**Figure 4:**
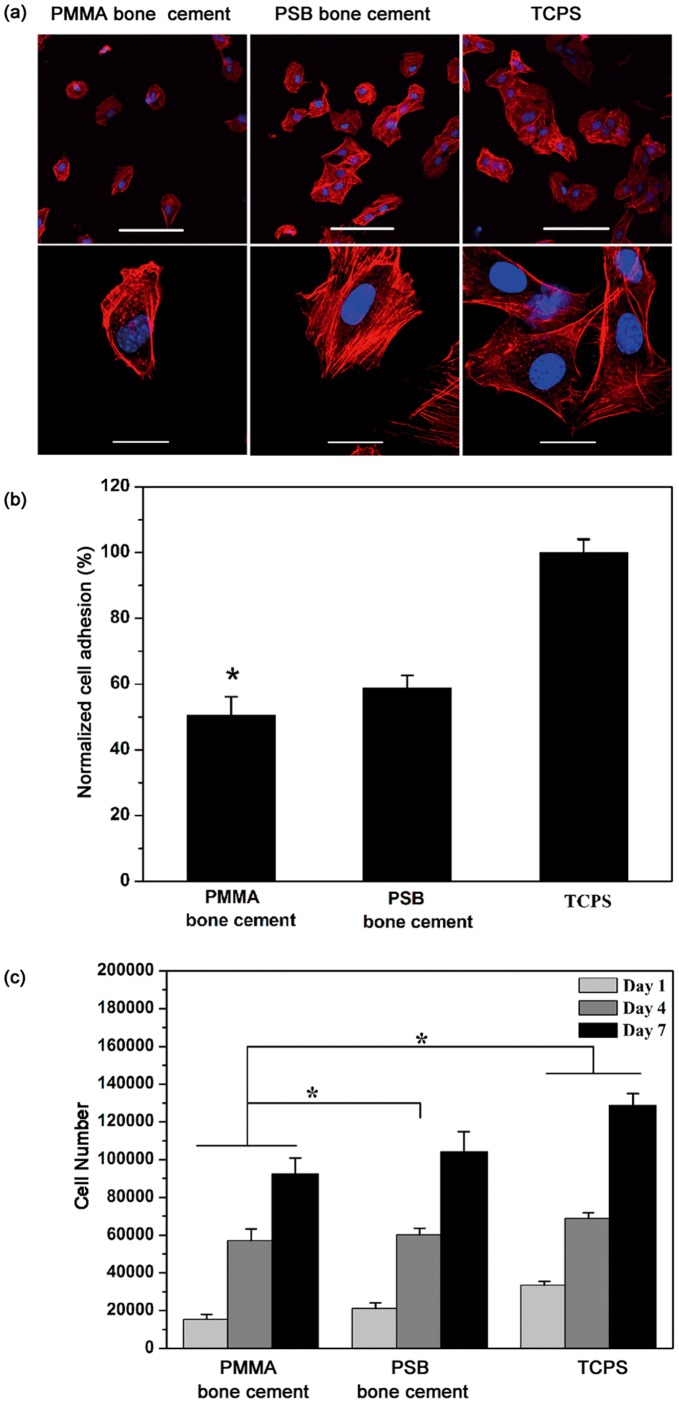
Cell adhesion and proliferation on disks of different bone cements. (a) Confocal images of F-actin and nuclei stained MC3T3-E1 cells cultured for 3 h on cement surfaces. Scale bar represents 100µm (top row) and 20 µm (bottom row). (b) Normalized cell adhesion 3 h post-seeding on the PMMA and PSB bone cement compared to cell-seeded TCPS as positive (+) control. Data are shown as mean ± standard deviation (n = 3).*, *p*<0.05 compared to TCPS and the PSB bone cement. (c) The number of MC3T3-E1 cells 1, 4, 7 days post-seeding on disks of the PMMA and PSB bone cement compared to cell-seeded TCPS as positive (+) control. Data are shown as mean ± standard deviation (n = 3).*, *P*<0.05 between two marked samples.

#### 6.2 Cell attachment, proliferation and differentiation

Disk samples of bone cement with dimensions of 10 mm in diameter and 1 mm in thickness were prepared, briefly submersed in 70% ethyl alcohol and allowed to dry under sterile conditions for all experiments. Tissue culture polystyrene (TCPS) plates were used as a positive control. The pre-incubated cell lines were placed on disk samples at densities of 5×10^4^, 2×10^4^ and 1.5×10^4^ cells/cm^2^ for the cell attachment, proliferation and differentiation tests. The time for MC3T3-E1 cell attachment on TCPS is usually 4 hours [24]. At 3 hours of adhesion the MC3T3-E1 cells had not yet completely adhered to the substrate and the difference of cell attachment could be found. Then attached cells were fixed using 3.7% formaldehyde solution, permeabilized with 0.2% Triton X-100 and stained using Rhodamine-phalloidin (Invitrogen, USA) and DAPI (Sigma–Aldrich, UK) for photographing using an Olympus FV1000 confocal microscope (Japan). The cell number attached on substrates was determined using MTS (methoxyphenyl tetrazolium salt) assay at this time. Normalized cell adhesion was calculated using the following equation: Normalized cell adhesion (%) = OD of test sample/OD of TCPS. The sample area for cell proliferation was only 0.785 cm^2^. The MC3T3-E1 cells will be in contact with each other and stop proliferation if the cells are incubated in culturing medium for over 7 days. Therefore, the MTS assay was used to evaluate cell numbers at 1, 4, 7 days. Cell differentiation from pre-osteoblasts to osteoblasts was determined as ALP activity. The enzyme ALP has been used as an indicator of osteoblastic activity for many years. The MC3T3-E1 cell is a clonal pre-osteoblastic cell line derived from newborn mouse calvaria [25]. The cells must be differentiated in osteogenic medium for over 7 days before a dramatic increase in the ALP activity can be measured. After culturing for 7 and 14 days in osteogenic medium, the ALP activity of cells on disk samples was measured using p-nitrophenyl phosphate (pNPP) (Sigma–Aldrich, UK) as described in a recent paper [26]. PNPP was converted into p-nitrophenol (pNP) in the presence of ALP at a rate that was proportional to the ALP activity. The production of pNP was determined using the absorbance that was measured at 405 nm wavelength using a micro-reader. The ALP activities were normalized to the total protein content.

**Figure 5:**
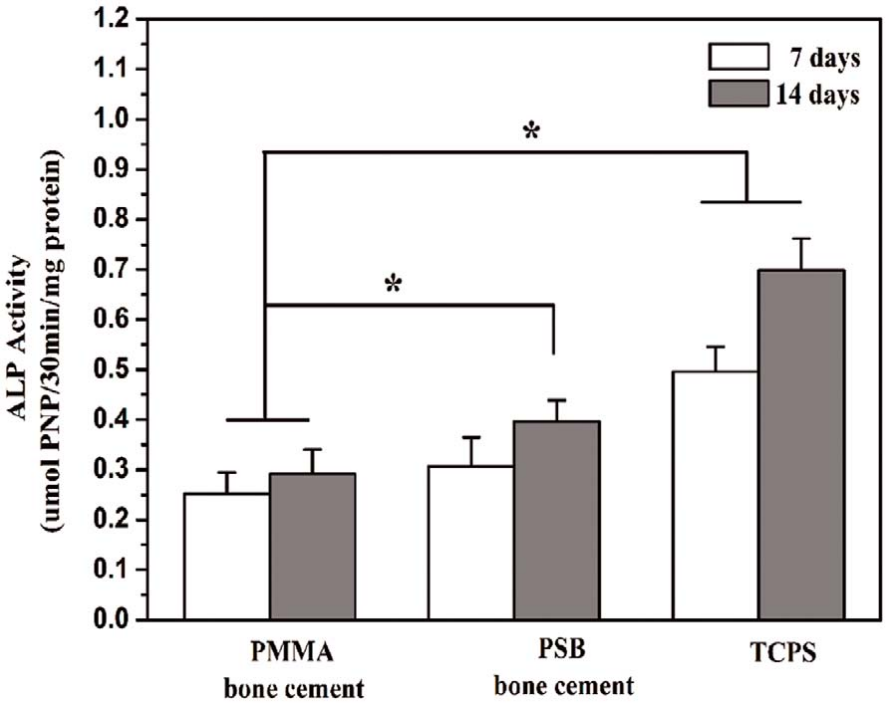
Cell differentiation on **disks of different bone cements.** ALP activities of MC3T3-E1 cells cultured on disks of the PMMA and PSB bone cement for 7 and 14 days, compared to cell-seeded TCPS as positive (+) control. Data are shown as mean ± standard deviation (n = 3).*, *P*<0.05 between two marked samples.

### 7. *In vivo* Test

For the *in vivo* animal tests, 44 adult female New Zealand white rabbits 5 months of age weighing between 3.5 and 4 kg were used. This study was carried out in strict accordance with the recommendations in the Guide for the Care and Use of Laboratory Animals of the National Institutes of Health. The animal protocol was approved by the Animal Care and Use Committee at Fourth Military Medical University (Permit Number: 08–269). A combination of ketamine hydrochloride (50 mg/kg, IM) and fentanile (0.17 mg/kg, IM) was used as the general anesthesia and 2% lidocaine with 1∶100000 epinephrine was injected as the local anesthesia. Hardened cylindrical specimens (10 mm in length and 6 mm in diameter) of the cement were prepared. The specimens were implanted into each medial femoral condyle. After surgery, the wounds were sutured with vicryl and penicillin (240,000 UI) was injected into the rabbits for 3 days. After 6 and 12 weeks, the rabbits were sacrificed with an overdose of sodium pentobarbital. For the micro-CT and histological analysis, harvested medial femoral condyles were fixed in a 10% neutral formaldehyde solution. Then the rabbit femur with hardened cement was imaged with three-dimensional microfocus computed tomography (micro-CT, eXplore Locus SP, GE, USA), at a voltage of 80 kVp and an electric current of 80 mA. The voxel size after reconstruction was 62.5 µm×62.5 µm×62.5 µm. To evaluate the *in vivo* resorption of the implanted materials, the residual material volume fraction (RMVF) was calculated as the ratio between the volume of residual material and the total volume of the materials. The porosity was quantified from the micro-CT data and calculated using the formula: Φ = V_V_/V_T_, where V_V_ is the volume of void-space and V_T_ is the total volume of material [27]. Based on the micro-CT results, the amount of bone ingrowth into the cement was quantified as the bone volume (BV) within the defined VOI (volume of interest) in each defect site. After the micro-CT scanning, the samples were embedded in methacrylate resin [28]. A total of 5 µm sections were obtained with a microtome (Microm-HM 350S, Thermo Fisher Scientific, USA). The sections were stained with Van Gieson’s Stain and examined with a light microscope (Nikon Microphot FXA). To investigate the strength of the implant–bone interface, push-out tests were conducted on a biomechanical test apparatus (SHIMADZU EHF-F01, Shimadzu Co., Kyoto, Japan). The test was performed following the procedure described in a previous report [29]. The maximum push-out force was determined and used to indicate the quality of the attachment to bone tissue.

**Figure 6:**
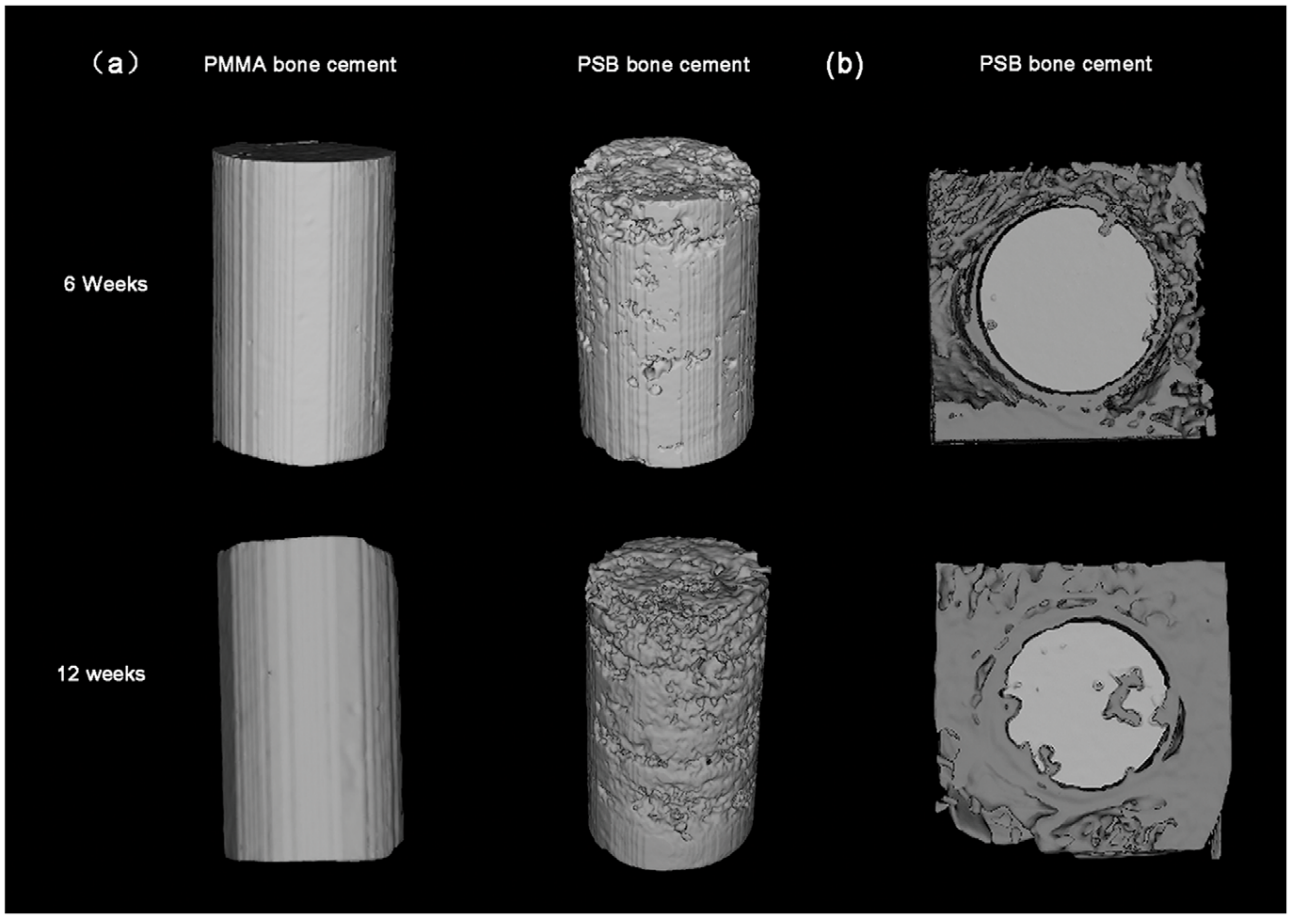
Tridimensional reconstruction using micro-CT analysis. (a) Residual material of the PMMA and PSB bone cement and (b) cross section images of rabbit femur after implantation for different periods.

**Figure 7:**
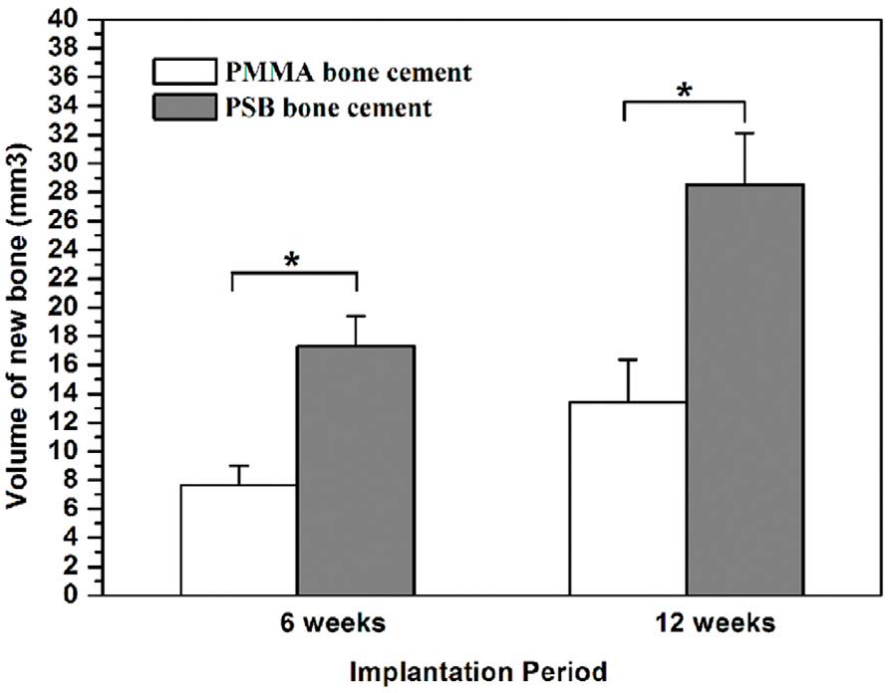
Quantitative analysis of new bone formation from micro-CT images. Data are shown as mean ± standard deviation (n = 5).*, *P*<0.05 between two marked samples.

### 8. Statistical Analysis

All statistical processing was completed using SPSS 16.0 (SPSS, Chicago, IL). A student’s t-test was used to analyze data between two groups. Differences between three groups were tested by a one-way analysis of variance (ANOVA) and differences between two groups were then compared using a Bonferroni post-hoc test. *P *values less than 0.05 were considered statistically significant. All errors are given as standard deviations.

## Results

### 1. Formulation and Setting Time of Cements Used

The CS particles displayed irregular shapes with the average diameter of about 200 µm. The glass particles consisted of numerous fine grains. The average diameter of the particles was about 40 µm. Table 2 shows the values of dough time and setting time of the cements used. The setting time of the PSB bone cement was longer than that of the PMMA bone cement (*p<*0.05). There was a small difference between the dough times of the PSB bone cement and those values of the PMMA bone cement.

**Figure 8:**
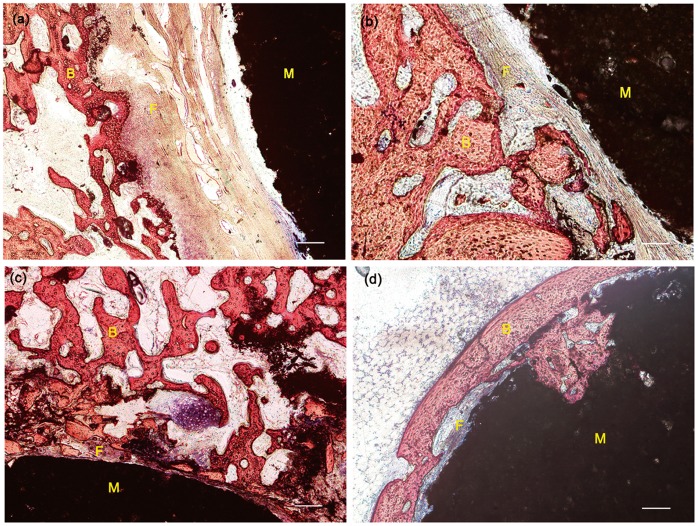
Histological morphologies of the interface between bone tissue and cement. (a, b) The PMMA and (c, d) PSB bone cement after implantation for 6 and 12 weeks, respectively. M: materials, B: bone, F: fibrous tissue, Arrow: bone ingrowth into macropores formed by the degradation of bone cement, bars = 100 µm.

### 2. Surface Morphologies of the PSB Bone Cement

SEM images of the PSB bone cement are shown in Fig. 1. The glass and CS particles were uniformly distributed in the polymeric matrix for dry cement samples. The space around the particles was present as the particles were not firmly integrated into PMMA matrix. However, the particles did not separate out. It’s already known that space around particles can increase the contact area between materials and body, thus accelerating the degradation rate of particles and promoting the formation of macropores [30].

**Figure 9:**
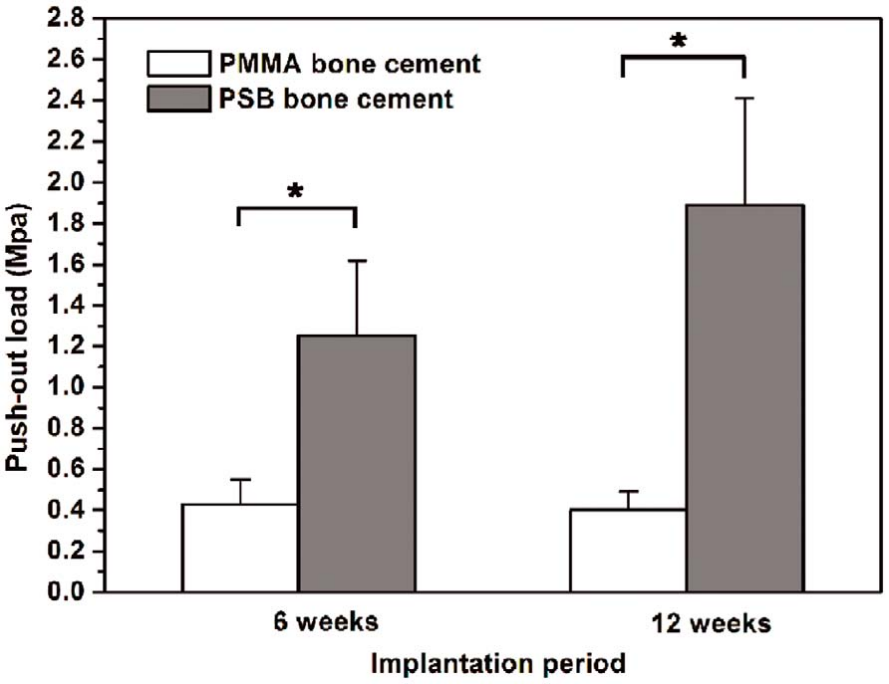
Results of push-out strength after implantation in rabbit femur. Data are shown as mean ± standard deviation (n = 6).*, *P*<0.05 between two marked samples.

### 3. Mechanical and Degradation Properties

Fig. 2 shows the compressive strength of the PSB and PMMA bone cement before and after degradation. It can be seen that the PSB bone cement had a lower initial compressive strength than the PMMA bone cement (*p<*0.05). Initially, the PSB bone cement had 80.31 Mpa in strength, which is in the range of that of bone (80–200 MPa). After 28 days degradation, the strength of the PSB bone cement decreased to 72.71 Mpa, which still meets the criterion (>70 MPa) listed in ISO 5833. In contrast, there was no significant decrease in the value of the PMMA bone cement after 28 days degradation.

### 4. Assessment of *in vitro* Bioactivity

The *in vitro* bioactivity of the PSB bone cement was investigated by soaking the samples in SBF. Fig. 3 shows SEM photographs of the surface of the cements before and after soaking in SBF for different periods. After 3 days, the surface changed considerably (as compared to the non-immersed specimens), but no deposits were visible even at high magnifications. Apatite crystals of needle-like morphology were observed on the surface of the cement sample soaked in SBF for 7 days. The EDS spectra of cement samples after immersion in SBF showed that silicon peaks almost disappeared and Ca and P peaks increased. The other elements (plus Cl from the solution) were also present in residual amounts on the surface.

### 5. Cell Attachment

Fig. 4a shows cytoskeletal and adhesion structures of MC3T3-E1 cultured for 3 h on disks of different bone cements. A significant difference in cytoskeleton organization was observed. The PMMA bone cement showed that F-actin in the seeded cells was disorganized while cells assumed a more or less spherical morphology. The PSB bone cement showed that cells developed well-organized F-actin bundles at their peripheries. The cell numbers attached on the disks of the PMMA bone cement at 3 h post-seeding were lower than those of the PSB bone cement (Fig. 4b) (*p<*0.05), suggesting that the PSB bone cement had better cell adhesion.

### 6. Cell Proliferation

MC3T3-E1 cell proliferation on bone cement disks was evaluated using MTS at different time points, as shown in Fig. 4c. It is evident that cell numbers increased continuously with the culture time, which indicates that all bone cements have good biocompatibility. The number of viable cells on the PSB bone cement was greater than that on the PMMA bone cement after 4 days culture (*p<*0.05), which suggests that the PSB bone cement was more beneficial for cell proliferation than the PMMA bone cement.

### 7. Cell Differentiation

Degree of differentiation of the MC3T3-E1 cells that were cultured in an osteogenic medium for 7 and 14 days on bone cements are shown in Fig. 5. The ALP activities of cells on the PSB bone cement were higher than those of cells on the PMMA bone cement after 14 days culture (*p<*0.05), suggesting that the glass particles in the PSB bone cement modulates preosteoblast cell differentiation.

### 8. Micro-CT Analysis

As shown in Fig. 6a, the 3D reconstruction images of residual material of the PMMA and PSB bone cement after implantation for 6 and 12 weeks were used to evaluate the *in vivo* resorption of the implanted cements. The areas and the volumes of the PSB bone cement can be seen to decrease with an increase of the implantation times. The dissolution of CS particles can result in macropore formation after 6 weeks of degradation. The pore size became greater when the bioglass particles close to the macropores degraded with time. It is clear to see that a porous surface construct was obtained after 12 weeks implantation. Furthermore, the porosity does not appear to be uniform. Obvious pore formation was found at the outer side of implant because the subcutaneous muscle layers generally contain large amounts of vascular tissues, which will accelerate the degradation process. However, no obvious pore formation was observed at the inner side of implant. Conversely, PMMA bone cement showed few observable changes even after 12 weeks of degradation. With increasing the implantation time from 6 to 12 weeks, the porosity of the PSB bone cement increased from 8.59±2.54% to 13.95±3.11%. In contrast, after 12 weeks of degradation, porosity was only 1.57±1.05% for the PMMA bone cement. Assessment of bone ingrowth into the implanted cements was also performed with micro-CT scanning (Fig. 6b). At 6 weeks, a small amount of newly formed bone was observed surrounding the PSB bone cement. And more extensive bone formations were observed throughout the cross-section of the bone cement at week 12 after implantation. Whereas only a few newly formed bones in the PMMA bone cement group appeared at the native bone margins and the defect periphery 12 weeks after implantation. The volume of new bone within the defect was calculated to evaluate the bone ingrowth more precisely (Fig. 7). The results indicated that the PSB bone cement contained a higher bone volume than the PMMA bone cement at both 6 and 12 weeks (*p*<0.05). The RMVF of the PSB bone cement decreased with the increase of the implantation time from 6 to 12 weeks, the RMVF decreased from 91.41±2.54% to 86.05±3.11%. Conversely, the RMVF of the PMMA bone cement remained 98.44±1.05% after 12 weeks implantation, which suggests the degradation rate of the PSB bone cement was much higher than that of the PMMA bone cement.

### 9. Histological Analysis

Bone-cement contact was confirmed using histological analysis at 6 and 12 weeks (Fig. 8). At 6 weeks after implantation, a dense fibrous layer was observed at the interface of the PMMA bone cement (Fig. 8a). On the other hand, direct contact with bone cement was observed at 6 weeks after implantation in the PSB bone cement. However, a thin fibrous layer was seen on those surface areas of the bone cement where PMMA was present (Fig. 8c). With the increase of the implantation period to 12 weeks, a thin fibrous layer was still observed at the interface of the PMMA bone cement, preventing bone contact (Fig. 8b). In contrast, for the PSB bone cement, obvious bone ingrowth into macropores formed by the degradation of bone cement occurred at 12 weeks after implantation (Fig. 8d).

### 10. Biomechanics

The strength of the implant–bone interface was examined using the push-out test after 6 and 12 weeks implantation (Fig. 9). It was obvious that the PSB bone cement had a higher value of push-out load than the PMMA bone cement at each implantation period. At week 12 after implantation, the push-out load of the PSB bone cement reached 1.89 Mpa, which was 4.7-fold higher than that of the PMMA bone cement.

## Discussion

Since the current PMMA bone cement used in total joint replacement is far from optimal, the bonding strength between bone and cement still needs to be enhanced. Many studies have been steadily conducted to improve the bonding strength at the interface. An early trial using Ceravital® particles reported tight bonding between the newly formed osseous tissue and the glass ceramic particles at the interface, but obtaining a bioactive composite cement with high mechanical properties was not achieved [31]. In this study, chitosan particles were chosen as porogen for the following reason: The addition of chitosan particles does not significantly reduce the mechanical property of the PMMA cement because no “macroscopic” weak links were present in the cement [32]. Therefore, the adequate mechanical property was achieved. Although the reduction in the compressive strength from 80 to 72 Mpa was observed after immersion in PBS for 4 weeks (Fig. 2), the value of strength still meets mechanical properties required by ISO 5833. Moreover, the mechanical properties would be enhanced by bone ingrowth under *in vivo* conditions.

One of the most important properties of cement is its setting time. The optimal time required is between 10 and 15 min [33]. If the setting time is too long, the surgeon must wait until he/she can close the wound [34]. Tsukeoka et al developed a bioactive PMMA cement through modification with gmethacryloxypropyltrimethoxysilane and calcium acetate, but the bone cement had a setting time of 18 min [29], which was beyond the range of clinical demands. In this study the PSB bone cement had a setting time of 12.7 min, which met clinical demands from a biological point of view.

We found the bioactivity was affected slightly by the low content of bioactive fillers. The results of SEM and EDS revealed that the formation of an apatite layer on the cement surface was confirmed until 7 days after soaking in SBF. Correspondingly, a histological examination also showed a thin fibrous layer on those surface areas of the bone cement where PMMA was present at week 12 after implantation (Fig. 8). This may be because of the low content of additives, which probably leaves less area of the bioactive fillers to react with the surrounding body fluid.

The *in vivo* degradation studies revealed that the formation of the porous surface structure was confirmed by the micro-CT analysis after implantation of the PSB bone cement in the rabbit femur defect. The *in vivo* resorption occurs with the increase of the implantation times. The residual material volume fraction of the PSB bone cement decreased to 86.05±3.11% at 12 weeks after implantation. The micro-CT results showed that the macropores seem to be formed by the degradation of the biodegradable CS particles, which was also observed in the histological images. Previous studies have shown that the pore size required for successful ingrowth of bone cells in orthopedics is at least 150 µm [35]–[38]. In the study, the average size of the macropores is about 200 µm, which is favorable for new bone ingrowth.

The findings of the present study suggest that the PSB bone cement resulted in a higher binding strength than the PMMA bone cement (Fig. 9). The results might be caused by the formation of the apatite layer and the osseointegration after implantation in the bony defect. The *in vitro* bioactivity using SBF clearly showed that the PSB bone cement had the ability to form apatite in the body environment. The apatite formation and release of calcium and phosphate ions were probably attributed to the increased degree of attachment, proliferation, and osteogenic differentiation of preosteoblast cells *in vitro*. A similar response was observed for osteoblast cells grown on PMMA/HA, where the proliferation of the cells on the composite was higher compared to PMMA after 8 days in the culture [39]. The formation of the apatite layer induced osteoconduction of the PSB bone cement through the surface reaction with surrounding body fluids *in vivo*. The micro-CT and histological analysis revealed that the newly formed bone was present around the PSB bone cement and direct bone apposition to the PSB bone cement was observed. In contrast, a dense soft tissue layer was seen at the interface of the PMMA bone cement. Quantitative analysis also showed that the volume of new bone in the PSB bone cement was remarkably higher than that in the PMMA bone cement (Fig. 7). The PSB bone cement stimulated more new bone formation than the PMMA bone cement on their surface during the implantation periods. More importantly, we observed obvious bone ingrowth into the PSB bone cement after implantation. The results indicated that the PSB bone cement possesses osseointegration properties, which is considered to be vital to firmly anchor the implant in place [40].

The results of the present study indicate that higher bonding strengths between bone and implant can be achieved with a porous surface modified bioactive bone cement. The low content of bioactive fillers resulted in proper handling characteristics and adequate mechanical properties, but slightly affected its bioactivity. The degree of attachment, proliferation and osteogenic differentiation of preosteoblast cells was also increased. Histological observation and micro-CT images showed that the modified bone cement exhibited osteoconductive properties and induced bone ingrowth into the porous surface structure. Our findings suggested a new bioactive bone cement for prosthetic fixation in total joint replacement. Further studies will attempt to investigate whether bone ingrowth into the bioactive bone cement will decrease the possibility of bone resorption in canine total hip arthroplasty.
